# Phylometabonomic Patterns of Adaptation to High Fat Diet Feeding in Inbred Mice

**DOI:** 10.1371/journal.pone.0001668

**Published:** 2008-02-27

**Authors:** Jane F. Fearnside, Marc-Emmanuel Dumas, Alice R. Rothwell, Steven P. Wilder, Olivier Cloarec, Ayo Toye, Christine Blancher, Elaine Holmes, Roger Tatoud, Richard H. Barton, James Scott, Jeremy K. Nicholson, Dominique Gauguier

**Affiliations:** 1 The Wellcome Trust Centre for Human Genetics, University of Oxford, Oxford, United Kingdom; 2 Division of Surgery, Oncology, Reproductive Biology and Anaesthetics, Department of Biomolecular Medicine, Faculty of Medicine, Imperial College London, London, United Kingdom; 3 Centre Européen de Résonance Magnétique Nucléaire à Très Hauts Champs à Lyon (CRMN Lyon) (FRE 3008), Laboratory of Chemistry (UMR 5185), Ecole Normale Supérieure de Lyon, Lyon, France; 4 Faculty of Medicine, Imperial College London, London, United Kingdom; University College Dublin, Ireland

## Abstract

Insulin resistance plays a central role in type 2 diabetes and obesity, which develop as a consequence of genetic and environmental factors. Dietary changes including high fat diet (HFD) feeding promotes insulin resistance in rodent models which present useful systems for studying interactions between genetic background and environmental influences contributing to disease susceptibility and progression. We applied a combination of classical physiological, biochemical and hormonal studies and plasma ^1^H NMR spectroscopy-based metabonomics to characterize the phenotypic and metabotypic consequences of HFD (40%) feeding in inbred mouse strains (C57BL/6, 129S6, BALB/c, DBA/2, C3H) frequently used in genetic studies. We showed the wide range of phenotypic and metabonomic adaptations to HFD across the five strains and the increased nutrigenomic predisposition of 129S6 and C57BL/6 to insulin resistance and obesity relative to the other strains. In contrast mice of the BALB/c and DBA/2 strains showed relative resistance to HFD-induced glucose intolerance and obesity. Hierarchical metabonomic clustering derived from ^1^H NMR spectral data of the strains provided a phylometabonomic classification of strain-specific metabolic features and differential responses to HFD which closely match SNP-based phylogenetic relationships between strains. Our results support the concept of genomic clustering of functionally related genes and provide important information for defining biological markers predicting spontaneous susceptibility to insulin resistance and pathological adaptations to fat feeding.

## Introduction

Reduction in the sensitivity to the peripheral and central actions of insulin (insulin resistance) is associated with several of the most frequent and prevalent human disorders, including type 2 diabetes mellitus, obesity and dyslipidemia [1], [2]. The etiology of these diseases is complex and involves genetic risk factors and environmental influences including sedentary lifestyle and dietary fat intake [3], [4], [5]. Numerous mechanisms contribute to insulin sensitivity regulation in several tissues (skeletal muscles, fat, liver) [6], thus making difficult pathophysiological investigations into the manifestation of insulin resistance in humans.

Rodent models of spontaneous or experimentally-induced insulin resistance are powerful tools for characterizing the physiological and molecular mechanisms involved in the onset and progression of diabetes and obesity. Targeted mutagenesis is extensively used to investigate the consequences of the inactivation of known genes on the regulation of insulin sensitivity *in vivo* [7]. High fat diet (HFD) feeding is also widely used in rodents to study the impact of dietary changes on the onset and progression of insulin resistance and obesity [8]. Strain-specific metabolic and hormonal adaptations to gene inactivation [9] or fat feeding [10], [11], [12], [13] in mice suggest that naturally-occurring genetic polymorphisms modulate spontaneous and experimentally-induced pathophysiological patterns. These models are therefore more relevant to polygenic obesity in human than models of gene inactivation.

Novel experimental systems and concepts have emerged to facilitate detection of linkages between genetic variants and disease phenotypes [14], [15], [16], [17]. Applying these strategies requires extensive knowledge of spontaneous and experimentally-induced phenotypic features in inbred founder strains [18], which can be maximized with consistent experimental designs aimed at standardizing general maintenance conditions, diet composition and duration of HFD feeding, assays and high density phenotype acquisition tools and technologies [19].

Metabonomics in particular is designed to identify in biological samples qualitative and quantitative changes in metabolic profiles [20] that underlie disease process and drug response in humans and model organisms [21]. Its role is central to the emerging field of nutrigenomics [22], which explores the genome-wide effects of nutrition, for isolating biomarkers characterizing dietary changes that can predict disease initiation and progression in different genetic makeups.

We have characterized the short-term consequences of fat feeding in five inbred strains (C57BL/6, 129S6, BALB/c, DBA/2, C3H) commonly used in genetic studies and as recipients for gene inactivation experiments (C57BL/6, 129S6) using an approach combining ^1^H NMR spectroscopy-based metabonomics and classical physiological and biochemical methods. Results from standardized multimodal phenotyping provide information on genetic backgrounds predisposing to spontaneous diabetes, and strain-specific patterns of metabolic and hormonal adaptations to fat feeding underlying mechanisms involved in the onset and progression of insulin resistance, diabetes and obesity.

## Materials and Methods

### Animals

Male mice of five inbred strains, C57BL/6JOxjr, BALB/cOxjr, DBA/2OlaHsd, 129S6/SvEvOxjr and C3H/He NHsd were used. These strains are referred as C57BL/6, BALB/c, DBA/2, 129S6 and C3H throughout the text. Colonies of C57BL/6, BALB/c and 129S6 were bred locally using stocks from the Jackson laboratory. DBA/2 and C3H mice were obtained from a commercial supplier (Harlan, UK). All mice were kept under standard maintenance conditions on 12h light/dark cycle. All experiments were carried out in accordance with national guidelines.

Mice were weaned at 21 days and caged in groups of 10. They were fed a normal carbohydrate (CHD) diet containing 5% fat, 19% protein and 3.5% fibre (S&K Universal Ltd, Hull, UK). At five weeks, mice were transferred on 40% high fat diet (HFD) containing 32% lard oil and 8% corn oil (Special Diet Services, Witham, UK) *ad libitum*. Strain- and age-matched groups remained on CHD throughout the experiment.

### Glucose tolerance and insulin secretion tests

Simplified intraperitoneal glucose tolerance tests (IPGTT) were performed in overnight fasted mice at 8 weeks of age (i.e. after 3 weeks of HFD feeding for the experimental group) as previously described [23]. Briefly, mice were anesthetized with an intraperitoneal injection of sodium pentobarbital (Sagatal, Rhône Mérieux, Harlow, UK) and a solution of glucose (2g/kg body weight) was injected intraperitoneally. Blood samples were collected from the tip of the tail vein before the injection and 15, 30 and 75 minutes afterward. Plasma samples were stored at -80°C until assayed for immunoreactive insulin (IRI). Cumulative glycemia (CumG) and insulinemia (CumIRI) were calculated as the increment of the values of plasma glucose and insulin, respectively, during the test. The ratio CumIRI/CumG was used as an index of insulin secretion. Incremental plasma insulin and glucose values above baseline, integrated over 75 min after glucose injection were used to calculate indices of insulin secretion (ΔIRI) and glucose tolerance (ΔG). The K parameter was calculated as the slope between glucose or insulin values at time points 15 and 75 minutes.

### Tissue sampling

Mice were allowed to recover for 4 days after the IPGTT. Body weight (BW) and length (BL) (distance from the tip of the nose to the base of the tail) were determined and BMI was calculated as BW/BL^2^. Mice were individually housed in metabolic cages for one overnight to determine food consumption. Digestible energy was calculated by multiplying the amounts of CHD and HFD eaten by 14 and 22.17, respectively. Following an overnight fast, mice were then killed by CO_2 _asphyxiation and a blood sample was collected on heparin by cardiac puncture. Blood was centrifuged and plasma was removed and stored at −80°C until lipid assay and ^1^H NMR metabonomic profiling. Retroperitoneal and epididymal fat pads (RFP and EPD, respectively) were collected and weighed. Adiposity indices (AI) were calculated as the ratio between RFP or EPD weight to BW (AI_RFP and AI_EPD, respectively).

### Analytical methods

Blood glucose concentration was determined during the IPGTT with a glucose meter (Accucheck, Roche Diagnostics, Welwyn Garden City, UK). Plasma IRI was determined on a 10 µl aliquot with an ELISA kit (Mercodia, Uppsala, Sweden). Plasma concentrations of total cholesterol (TC), high density lipoprotein cholesterol (HDL), low density lipoprotein cholesterol (LDL) and triacylglycerol (TG) were determined using diagnostic enzymatic/colorimetric kits (ABX, Shefford, UK) on a Cobas Mira Plus automatic analyser (ABX, Shefford, UK).

### Metabolic profiling in plasma samples

^1^H NMR spectra were acquired at 600.22 MHz on a Bruker Avance-600 spectrometer (Bruker, Coventry, UK) using 100 µl of plasma diluted in 400 µl of a 9g/l saline solution (20% ^2^H_2_O/H_2_O v/v). Standard monodimensional spectra were acquired using a selective presaturation pulse sequence for water signal suppression as described elsewhere [24]. For each acquisition, 128 transient free induction decays (FID) were collected into 32,768 data points using a spectral width of 20 ppm. The FIDs were convoluted with an exponential weighting function corresponding to a line broadening of 0.3 Hz before Fourier transform. The NMR spectra were corrected for phase and baseline distortions using in-house software and referenced to the glucose α-anomeric signal (δ5.23) [24]. The ^1^H NMR spectra were imported as full-spectral resolution data [25]. Each variable reflects the exact NMR datapoint to which it was associated. To remove the effects of variation in the suppression of the water resonance, the region (δ4.5–5.0) was discarded. The data were normalized by using a noise variance normalisation. The final dataset was then mean-centred prior to analysis [25].

### Statistical methods

Phenotypic data collected from the animals were analyzed using the SPSS version 12.0 statistical package. The univariate General Linear Model (GLM), which allows the selection of covariates to account for variance that is not due to the dependent variable, was used to analyse phenotypes. Fisher's LSD and Tamhane's T2 post hoc tests were used according to Levene's test for equality of variance in order to assess differences between the strains on carbohydrate diet and high fat diet. Bivariate Pearson pairwise correlations were obtained using R package ‘rma’ and plotted in the form of a correlation matrix using the following phenotypes: glycemia, insulinemia, homeostasis model assessment of insulin resistance (HOMA), plasma lipids, body weight, BMI, AI_RFP and AI_EPD. Principal component analysis (PCA) was used to visualize the data and for quality control again using the phenotypes mentioned in obtaining the Bivariate Pearson pairwise correlations.

### Multivariate pattern recognition of ^1^H NMR spectra

The O-PLS algorithm derives from the basic partial least squares (PLS) regression (15). In discriminant analysis version, it explains maximum separation between class samples ***y*** (1 dummy variable for a two-class discrimination in our case) using the NMR data ***X***, by decomposing the covariation matrix (***y*** ^T^ ***X***) into 1 predictive O-PLS component and several orthogonal signal correction (OSC) components [26]. Further details on O-PLS standard implementation in metabonomics are given in ref [25]. The model coefficients locate the NMR variables associated to a specific class in ***y***. The model coefficients were then back-scaled in order to enhance interpretability of the model: in the coefficient plot, the intensity corresponds to the mean-centred model (variance) the colour-scale derives from the unit variance-scaled model (correlation).

### Hierarchical clustering trees (HCTs)

Hierarchical clustering was used to generate phylogenetic and phylometabonomic trees based on SNP and NMR data, respectively. HCTs are measures similarity or dissimilarity between observations computed from the data matrix. Dissimilarity coefficients are symmetric, i.e. ***d(A,B) = d(B,A)*** and non-negative i.e. ***d(A,A) = 0*** and can be displayed as dendrograms. The single-linkage method applied here uses the maximum Euclidean distance between two profiles (SNP genotypes or ^1^H NMR metabotypes) as the dissimilarity measure. SNP data were coded as Strain Distribution Patterns (SDP) prior to building the phylogenetic trees. Average ^1^H NMR spectra for each strain and diet condition were used to build phylometabonomic classifications.

## Results

### Global impact of genetic differences on metabolic and hormonal regulations

**Figure 1:**
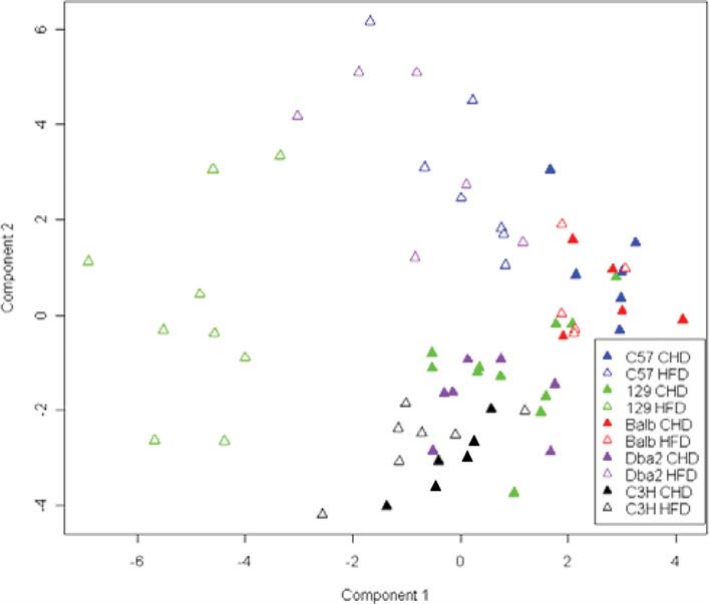
Principal component analysis (PCA) in inbred mice fed control (CHD) or high fat diet (HFD). PCA was carried out with classical physiological data (glucose tolerance, insulin secretion, BW, BMI and adiposity indices) collected in eight week old mice fat fed for three weeks and controls. Prior to PCA, phenotypic variables were mean centered and reduced to a variance of 1. Phenotypes contributing to separation between models are described in Figure S1.

We used PCA of classical metabolic and hormonal variables in HFD- and CHD-fed mice to underline strain and diet latent factors underlying global phenotypic variations (Fig 1). Principal component (PC) 1 highlighted differences between two main groups on CHD (C3H, DBA2, 129S6 and C57BL/6, BALB/c). Whist PC1 also allowed the separation of HFD-fed and CHD-fed mice of the 129S6 strain, PC2 predominantly accounted for fat feeding effects in DBA/2 and C57BL/6 mice. Glycemia and adiposity indices were the top ranking phenotypes responsible for the separation between HFD-fed and CHD-fed 129S6 mice, whereas insulin data contributed to the separation of fat fed and CHD-fed groups identified by PC2 (Figure S1). In both BALB/c and C3H, PCA resulted in clustering the HFD- and CHD-fed groups, suggesting that fat feeding had little impact on the phenotypes analyzed (Fig 1). Individual phenotypes were analysed further to refine these apparent strain-specific patterns of adaptation to HFD feeding.

### Body growth, adiposity and lipid metabolism

When fed CHD, mice of the strains C3H and C57BL/6 were significantly heavier than DBA/2 and BALB/c mice (p<0.001) (Table 1). C3H mice consumed significantly more CHD than the other four strains. Adiposity indices were significantly greater in DBA/2 mice than in the other mouse strains (p<0.001). After three weeks of HFD feeding, BW, BMI and adiposity indices were consistently significantly increased in 129S6 and C3H strains when compared to age-matched CHD-fed controls. These variables were unaffected by HFD in DBA/2 mice. The other two strains showed an intermediate pattern of adaptation to fat feeding characterized by increased BW and BMI without changes in adiposity indices in C57BL/6 or unchanged BW and BMI, despite significantly increased adiposity indices (+>130%) in BALB/c. The latter strain was the only model to exhibit increased food energy consumption on HFD (+14%). HFD induced a significant reduction of food energy consumption in C57BL/6 (−45%), C3H (−35%) and DBA/2 (−25%) mice.

**Table 1:** Body weight (BW), body mass index (BMI), adiposity indices (AI) and digestible energy in 8 week old mice of different inbred strains fed carbohydrate diet (CHD) or high fat diet (HFD) for three weeks.

	Carbohydrate diet					High fat diet				
	C57BL/6	129S6	BALB/c	DBA/2	C3H	C57BL/6	129S6	BALB/c	DBA/2	C3H
BW (g)	23.8±0.2 (83)	23.1±0.2 (93)	22.3±0.2 (128)	22.8±0.2 (136)	24.6±0.3 (40)	24.8±0.2 (87)*	25.4±0.3 (84)**	22.8± 0.21 (64)	23.8±0.3 (149)	26.9±0.3 (40) ***
BMI (g/cm^2^)	2.69±0.03 (83)	2.56±0.02 (91)	2.58±0.03 (117)	2.84±0.02 (136)	2.72±0.04 (40)	2.89±0.03 (79) ***	2.92±0.03 (83) ***	2.65±0.04 (64)	2.91±0.02 (148)	2.90±0.03 (40) **
AI_EPD (x1000)	9.0±0.6 (16)	15.4±0.9 (12)	6.3±0.8 (7)	17.4±1.3 (15)	9.0±0.7 (10)	8.6±1.1 (8)	24.2±1.4 (12) **	15.6±1.6 (8) *	22.1±2.2 (12)	17.8±1.8 (10) *
AI_RFP (x1000)	1.29±0.14 (16)	4.03±0.23 (12)	1.21±0.13 (7)	5.50±0.34 (15)	2.91±0.29 (10)	1.61±0.17 (8)	7.43±0.38 (12) ***	2.77±0.28 (8) *	7.66±1.06 (12)	5.20±0.45 (10) *
Digestible energy (mJ/Kg)	51.5±4.9 (22)	45.5±2.0 (34)	57.6±2.4 (23)	48.3±2.0 (50)	68.6±1.8 (28)	28.3±3.7 (24) *	39.0±1.9 (36)	65.8±4.2 (24)	36.2±2.3 (68) **	44.0±4.8 (28) **

Retroperitoneal and epididymal fat pads were used for AI calculation. Data are means±SEM. Number of mice is reported in parentheses. Differences between groups were assessed by Fisher's LSD and Tamhane's T2 post hoc tests. *p<0.05, **p<0.01, ***p<0.001; significant differences between HFD and CHD mice of the same strain.

**Figure 2:**
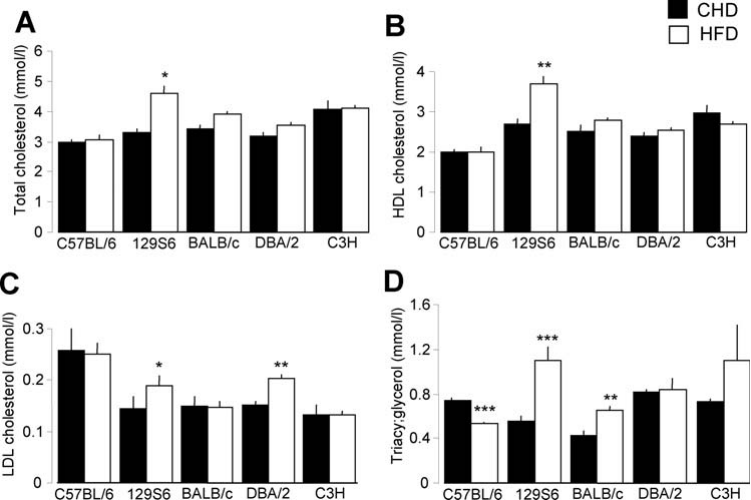
Effects of high fat diet (HFD) on plasma lipids in inbred mouse strains. The effects of fat feeding on total cholesterol (T-C) (A), HDL-C (B), LDL-C (C) and triacylglycerol (D) are shown. Values are expressed as means±SEM. Data were obtained from over 8 mice of each group. Differences between groups were assessed by Fisher's LSD and Tamhane's T2 post hoc tests. *p<0.05, **p<0.01, ***p<0.001, significantly different to CHD fed mice of the same strain.

Plasma levels of total cholesterol (TC) and LDL-C were similar in the 5 strains on CHD, whereas HDL-C was significantly lower in C57BL/6 than 129S6 and DBA/2 mice and plasma TG levels were significantly more elevated in DBA/2 and C57BL/6 than 129S6 and BALB/c mice (Fig. 2). Fat feeding in 129S6 mice induced a statistically significant general raise of plasma TC, HDL-C, LDL-C and TG. Some of these effects (TC, HDL-C) were specific to this strain. The effect of HFD on increased plasma LDL-C was also observed in DBA/2 mice. Plasma TG levels were increased by HFD in BALB/c and paradoxically reduced by HFD in C57BL/6.

Overall these individual phenotype features in the mouse models are consistent with the proposed complex pattern of adaptations to fat feeding derived by PCA.

### Glucose homeostasis and insulin secretion

**Figure 3:**
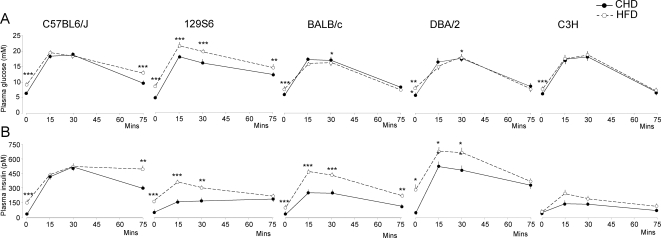
Glucose tolerance (A) and glucose induced insulin secretion *in vivo* (B) in inbred mouse strains. Data are derived from 8 week old mice fed CHD or HFD for three weeks. Data shown are mean±SEM (n = 9 to 86 mice per group). Differences between groups were assessed by Fisher's LSD and Tamhane's T2 post hoc tests. *p<0.05, **p<0.01, ***p<0.001, significantly different to CHD fed mice of the same strain.

**Figure 4:**
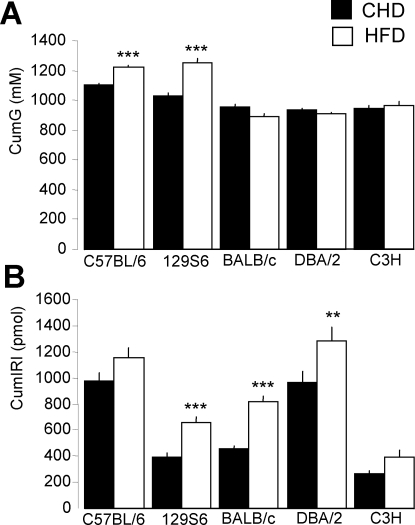
Effects of high fat diet (HFD) on cumulative glycemia (A) and insulinemia (B) in mice. Values of cumulative glycemia (CumG) and insulinemia (CumIRI) are expressed as means±SEM. Data are derived from 15 to 40 mice per group. Differences between groups were assessed by Fisher's LSD and Tamhane's T2 post hoc tests. ***p<0.001, significantly different to CHD fed mice of the same strain.

Results from glucose tolerance and *in vivo* insulin secretion testing were used to identify strain differences and similarities in the regulation of these processes when mice are fed CHD or HFD. On CHD, C57BL/6 mice had significantly higher fasting glycemia (P<0.001) than the other strains (6.2±0.1 mM in C57BL/6 when compared to 4.4±0.1 mM in 129S6, 5.6±0.1mM in BALB/c, 5.4±0.1 mM in DBA/2 and 5.8±0.2 mM in C3H) (Fig. 3A). Fasting insulinemia was similar in the 5 strains (Fig. 3B). All CHD fed strains showed similar glycemic patterns during the IPGTT (Fig. 3A), but higher cumulative glycemia (CumG) during the IPGTT in C57BL/6 mice than in the other strains (P<0.01) suggested relative glucose intolerance in this strain (Fig. 4A). Mice of the C57BL/6 and DBA/2 strains showed greater glucose induced insulin secretion capacity than the other strains (Fig. 3B), as evidenced by the higher values of cumulative insulin (CumIRI) in the former strains (Fig. 4B).

When fed HFD, all strains became significantly hyperglycemic (Fig. 3A). Glucose intolerance assessed by CumG values developed only in C57BL/6 and 129S6 mice (Fig. 4A) and appeared more marked in 129S6 mice (Fig.3A). Fasting hyperinsulinemia developed in all strains but C3H (Fig. 3B). Mice of the 129S6, BALB/c and DBA/2 strains showed significantly enhanced glucose induced insulin secretion *in vivo* (Fig. 4B).

Overall, even though the strains exhibited specific patterns of regulations of glucose homeostasis and insulin secretion, three weeks of HFD feeding generally induced profound hyperglycemia and hyperinsulinemia. Strongly significant enhancement of fasting insulinemia and insulin secretion induced by HFD in 129S6, BALB/c and DBA/2 had little effect on glucose tolerance (BALB/c, DBA/2) or was associated with glucose intolerance (129S6), suggesting insulin resistance in these strains.

### Phenotype correlations

**Figure 5:**
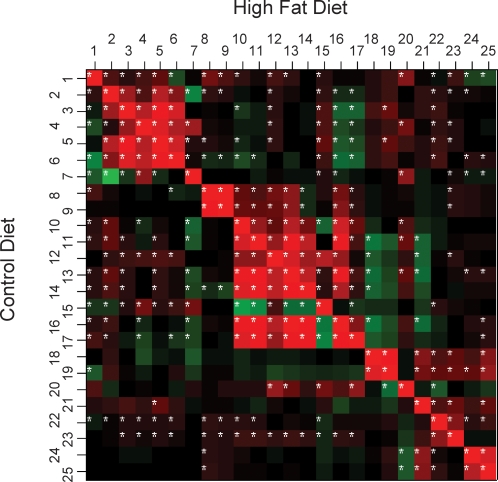
Correlation matrices plotted for all strains on HFD and CHD diets. Negative and positive correlations are shown for each pair of phenotypes in green and red, respectively. Bivariate Pearson pairwise correlations were derived from the R package ‘rma’. Asterisks indicate statistically significant correlations. Correlation plots for individual strains are shown in Figure S2. 1, IPGTT glycemia time 0; 2, IPGTT glycemia time 15; 3, IPGTT glycemia time 30; 4, IPGTT glycemia time 75; 5, IPGTT Cumulative glycemia (CumG); 6, IPGTT glucose tolerance index (ΔG); 7, K glucose parameter; 8, Homa index; 9, IPGTT insulinemia time 0; 10, IPGTT insulinemia time 15; 11, IPGTT insulinemia time 30; 12, IPGTT insulinemia time 75; 13, IPGTT Cumulative insulinemia (CumIRI); 14, IPGTT insulin secretion index (ΔIRI); 15, K insulin parameter; 16, IPGTT insulin secretion index (CumIRI/CumG); 17, IPGTT insulin secretion index (ΔIRI/ΔG); 18, plasma total cholesterol; 19, plasma high density lipoprotein cholesterol; 20, plasma low density lipoprotein cholesterol; 21, plasma triacylglycerol; 22, body weight; 23, body mass index; 24, adiposity index (retroperitoneal fat); 25, adiposity index (epididymal fat).

To investigate relationships between phenotypes, individual correlations were calculated. When phenotype data from all strains were analyzed, the highest correlations in both fat fed and CHD fed groups were, as expected, for intra-experimental measurements, i.e. between glucose tolerance data and between insulin secretion data and between adiposity indices and plasma TG levels (Fig. 5). Significant correlations between plasma lipids and insulin secretion data, body weight, BMI and adiposity indices were observed only in the fat fed group. Correlations between fasting insulinemia and insulin secretion data were also only detected specifically in the fat fed group.

This pattern of phenotype relationships was generally conserved when individual strains were tested (Figure S2). Strain-specific phenotype relationships included significant correlations between glucose tolerance and insulin secretion (CHD-fed BALB/c), body weight (CHD-fed DBA2 and C3H) or adiposity indices (CHD-fed C3H). Significant strain-specific correlations were also observed for fasting insulinemia and adiposity indices (CHD-fed C57BL/6) or plasma TG (CHD-fed 129S6). In HFD-fed mice, lack of relationship between fasting insulin and insulin secretion was specific to DBA/2 and C3H.

### Plasma ^1^H NMR metabonomic profiling

**Figure 6:**
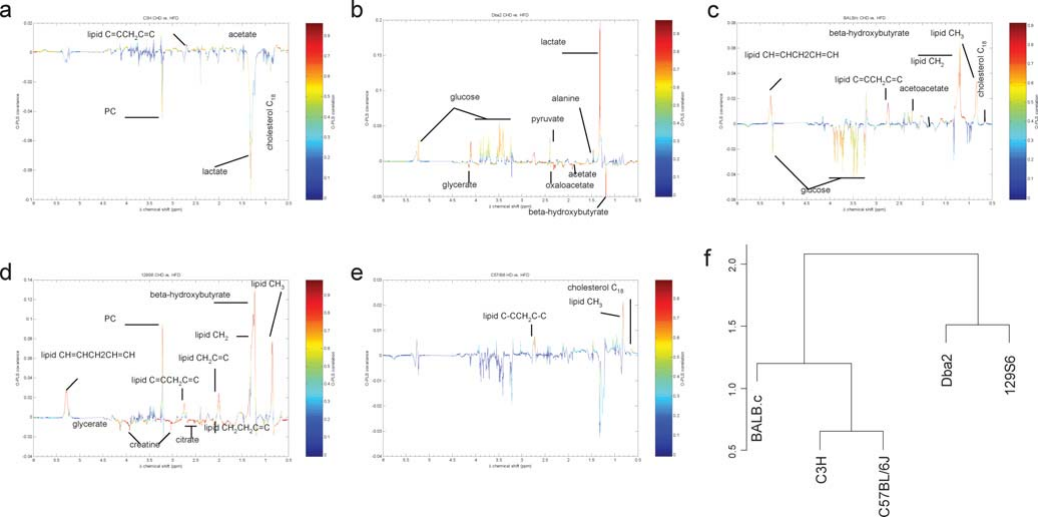
^1^H NMR metabonomics derived metabotypes supporting classification of 5 inbred mouse strains. O-PLS-DA model coefficients were used for differential analysis between diets and strains in C3H (a), Dba2 (b), BALB/c (c), 129S6 (d) and C57Bl/6 (e). Global clustering analysis (f) uses a scale which corresponds to the Euclidean distance between strains and depends on the dynamic range of the variables.

To identify strain-specific metabolic changes in models predisposed or resistant to spontaneous or diet-induced relative glucose intolerance, adiposity or hyperlipidemia, ^1^H NMR spectral data from CHD- and HFD-fed mice were independently analyzed using O-PLS-DA (Fig 6 and Figure S3). Goodness of fit statistics corresponding to each model is given in Table 2. In the CHD-fed group the most significant metabolic alterations that contributed to strain separation included increased levels of glucose in C57BL/6, citrate and pyruvate in 129S6, and lactate, lipids and phosphocholine (PC) in C3H (Figure S3). Reduced concentrations of lipids, choline and phosphocholine (PC) were specific to C57BL/6, and decreased levels of glucose, lactate and oxoglutarate to DBA/2. Even though several strain-specific metabolic changes were conserved in HFD-fed mice (e.g. lipids, choline, PC in C57BL/6 and oxoglutarate in DBA/2), adaptations to HFD also involved altered levels of different sets of metabolites specific to C57BL/6 (lactate), 129S6 (PUFAs, PC), BALB/c (β-hydroxybutyrate), DBA/2 (alanine, pyruvate), and C3H (phenylacetate, creatine).

**Table 2:** O-PLS-DA model goodness of fit statistics.

Model/Goodness of fit	R^2^X	R^2^Yhat	Q^2^Yhat
**C3H (CHD vs. HFD)**	0.85	0.89	0.77
**Dba2 (CHD vs. HFD)**	0.76	0.95	0.90
**BALB/c (CHD vs. HFD)**	0.65	0.95	0.78
**129S6 (CHD vs. HFD)**	0.87	0.96	0.90
**C57 (CHD vs. HFD)**	0.85	0.87	0.71
**CHD (5 strains)**	0.96	0.91	0.78
**HFD (5 strains)**	0.98	0.94	0.84

For each O-PLS-DA model represented in Fig 6 and Fig SI 3, R^2^X, R^2^Y_hat_ and Q^2^Y_hat_ are calculated according to Cloarec et al. [25].

Combined analysis of genetic and dietary factors showed that group separation was characterized by differential regulation of the concentrations of lactate (C3H, DBA/2), glucose (BALB/c, DBA/2) and PC (C3H, 129S6) (Fig. 6). Increased PC level remained significantly associated with 129S6. BALB/c and 129S6 mice shared conserved metabolic patterns, including increased concentration of lipids and β-hydroxybutyrate.

**Figure 7:**
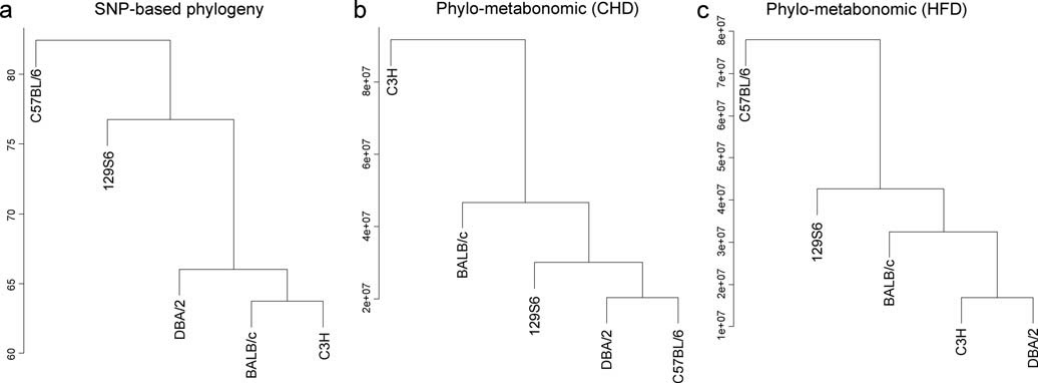
Genetic and functional relationships between inbred mouse strains. Phylogenetic relationships between the five strains used in the study (a) derive from published data of over 10,000 SNP [27] available at http://gscan.well.ox.ac.uk/gs/strains.cgi. ^1^H NMR metabonomic data were used for clustering analysis of inbred strains fed carbohydrate diet (CHD) (b) or high fat diet (HFD) (c). The scale corresponds to the Euclidean distance between strains used to grow the clustering tree, which is dependent of the dynamic range of the variables.

To test the existence of a relationship between genetic variability and strain-specific metabolic patterns, we used datasets of over 10,000 SNP recently produced in these five strains [27] (http://gscan.well.ox.ac.uk/gs/strains.cgi) to derive phylogenetic clustering, which we compared to ^1^H NMR-based metabotypic clusters (Fig. 7). Resulting phylometabonomic patterns reflect natural and diet-induced metabolic variability between strains. These patterns were clearly different in CHD- and HFD-fed groups (Fig. 7) and when both strain and dietary conditions were used for clustering analysis (Fig. 6). Metabolic patterns in HFD-fed mice closely match phylogenetic relationships between the strains tested. The apparent metabolic refocusing in HFD-fed animals underlines the importance of diet-reactive genetic polymorphisms in these animals, *i.e.* nutrigenomic predisposition.

## Discussion

We have used a consistent series of protocols and standardized methods and experimental conditions to comprehensively assess the short-term metabolic and hormonal consequences of fat feeding in five genetically well-characterized inbred mouse strains. PCA identified the strongest spontaneous and diet-induced strain differences and similarities, which were refined by physiological and biochemical studies. Our findings highlight the impact of genetic differences on the regulation of insulin secretion and action, and the complex pathophysiological patterns of adaptations to HFD feeding, which underlie nutrigenomic predispositions to insulin resistance and obesity.

The robustness of our dataset lies in the application of strictly standardized and synchronized phenotyping which minimized environmentally-induced phenotype variations and allowed reliable comparisons between strains and conditions for multiple phenotypes. Results from physiological tests generally confirm published evidence of poor glucose homeostasis that C57BL/6 mice spontaneously exhibit [11], [12], [23] and high insulin secretion capacity in C57BL/6 and DBA/2 strains relative to BALB/c and C3H [23]. Comparisons with other mouse phenotype datasets, often aimed at identifying contrasting biological variables in a pair of strains used in a genetic cross, remain difficult due to differences in genetic backgrounds, technical assays and maintenance conditions that inevitably induce phenotype variations, even in self contained experiments [28]. Of relevance to our study, the mutation in the nicotinamide nucleotide transhydrogenase gene which causes insulin deficiency in C57BL/6 mice [29] was not found in our C57BL/6 colony (data not shown).

Fat feeding introduced an additional level of complexity in strain-specific metabolic and hormonal patterns. Consensus features of adaptation to HFD in all strains tested were hyperglycemia and hyperinsulinemia. Fat fed mice exhibited some evidence of insulin resistance as increased insulin secretion was associated with glucose tolerance which was either deteriorated (C57BL/6, 129S6) or unchanged (BALB/c, DBA/2, C3H), and adiposity indices either increased (129S6 BALB/c, C3H) or were unchanged (C57BL/6, DBA/2). Only C57BL/6 and 129S6 strains developed marked glucose intolerance and the latter exhibited the most severe phenotypic alterations characterized by increased body weight, adiposity indices and plasma lipid levels.

Although our results largely agree with data from other studies in HFD-fed mice [13], [30] we noted inconsistencies that may be caused by above outlined factors and differences in HFD composition and fat feeding duration. Results from our laboratory showed that phenotype alterations in 129S6 fed the same 40% HFD were more profound when fat feeding was prolonged (15 weeks) and combined obesity, hyperlipidemia, enhanced insulin secretion, glucose intolerance and fatty liver [31], [32]. In contrast HFD-fed BALB/c mice maintained in identical conditions showed both enhanced insulin secretion and markedly improved glucose tolerance, suggesting normal or enhanced insulin sensitivity which may prevent fatty liver in this strain [31]. In addition, increased severity of fatty liver and obesity phenotypes in 129S6 mice fed a 40% HFD deficient in choline illustrates the strong impact of apparently minor alterations in diet composition on disease progression [31].

Correlative relationships between multiple phenotypes in fat fed and control mice underlined strain-specific and conserved features of susceptibility or resistance to diet induced obesity. Reduced insulin secretion capacity in CHD-fed 129S6, BALB/c and C3H mice relative to C57BL/6 and DBA/2 did not correlate with HFD-promoted glucose intolerance which develops more prominently in C57BL/6 and 129S6 mice. Interstrain differences in insulin secretion in both fat fed and control mice did not correlate with patterns of adiposity indices either. The integration of metabonomic profiling data from fat fed and control mice in these highly heterogeneous pathophysiological patterns of response to standardized HFD feeding potentially provides tools for identifying predictive biomarkers associated with specific disease traits determined by different genetic backgrounds. For example, altered metabolism of choline and lipids appear to be central features of 129S6 which exhibits the strongest susceptibility to diet induced obesity, diabetes and fatty liver as previously shown [31]. In the context of nutrigenomics [22], metabolic profiles are particularly important as they accurately document metabolic endpoints of gene expression regulation at the organism level, which in contrast to transcriptomics do not require prior knowledge of organ-specific altered mechanisms [33].

Results from our study show that metabolites derived from metabonomic profiles are associated with several types of strain-specific disease signatures associated with HFD response, including ketosis in BALB/c and lactic acidosis in C3H and DBA/2. Evidence of dyslipidemia patterns, including increased PC level remained significantly associated with 129S6, as previously described in a gut microflora modulation context [31]. Mice of the BALB/c and 129S6 strains shared conserved metabolic patterns, including increased concentration of PUFA lipids and increased ketosis (β-hydroxybutyrate and acetoacetate).

Testing causal relationships between metabotypes, pathophysiological traits and genetic polymorphisms in mice require similar investigations in classical genetic crosses or in hybrids of the heterogeneous stock [16] and the collaborative cross [15] which both derive from strains tested in this study. We have recently shown that untargeted ^1^H NMR metabonomic quantitative traits can be mapped to the genome in an F2 cross, and that a proportion of these co-localise with loci linked to pathophysiological traits [34]. The definition of metabonomic clusters of ^1^H NMR spectral data from strains of mice fed CHD and HFD, which reflect the role of metabolic and genetic variations in strain separation, indicates that metabonomic QTL mapping is possible in mice and requires strictly standardized experimental conditions in cohorts of hybrid mice. SNP-based phylogenetic relationships between strains were generally consistent with established mouse genealogy data [35], [36], [37] and closely match phylometabonomic patterns in fat fed mice. These data highlight the important role of genetic variants on metabolic regulations and support the involvement of metabolic refocusing in HFD-fed mice. This apparent concordance between phylogenetic trees, which estimate haplotype conservation and divergence between strains, and diet-specific phylometabonomic relationships supports the concept of genomic clustering of functionally related genes [38].

Diet-induced changes in phylometabonomic patterns show that murine plasma metabolome is strongly influenced by environmental factors, resulting in a context-dependent modulation of the genome-encoded traits. Such metabotypic variation may also involve non-genomic influences including cooperative interactions between microbial and mammalian metabolism, which have emerged from metabonomic-based studies in humans and animal models [39]. We have demonstrated in fat fed mice that transgenomic effects result in strain-specific changes in the abundance of metabolites processed by the gut microflora (e.g. choline as discussed above), which contribute to fatty liver predisposition and severity, and are therefore directly relevant to disease susceptibility [31].

### Summary

We report here the breadth of metabolic and hormonal patterns of the spontaneous and nutrigenomic susceptibility to insulin resistance, obesity and diabetes in inbred mouse strains. Results from extensive phenotyping in different strains demonstrated the varied pathophysiological consequences of fat feeding on intermediate phenotypes, rather than the development of a single phenotype entity unifying glucose intolerance, hyperinsulinemia, obesity and hyperlipidemia. Metabonomic biomarkers of adaptation to fat feeding represent molecular targets for further studies designed to test their power to predict susceptibility or resistance to spontaneously occurring and experimentally induced disease patterns.

## Supporting Information

Figure S1 (0.11 MB TIF)

Figure S2 (0.62 MB TIF)

Figure S3 (0.14 MB TIF)
