# Supplementary figures and images for: Phylometabonomic Patterns of Adaptation to High Fat Diet Feeding in Inbred Mice
Source: PLoS One. 2008 Feb 27;3(2):e1668. doi: 10.1371/journal.pone.0001668 (PMC2244706; doi:10.1371/journal.pone.0001668)

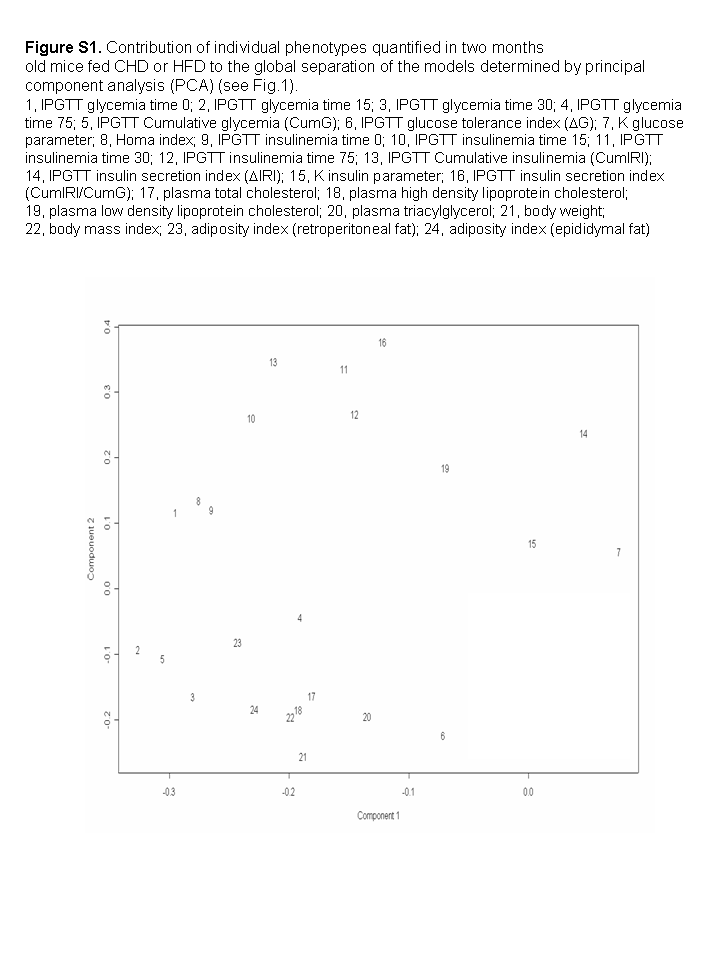

Supplement: Figure S1 — (0.11 MB TIF) [file pone.0001668.s001.tif]

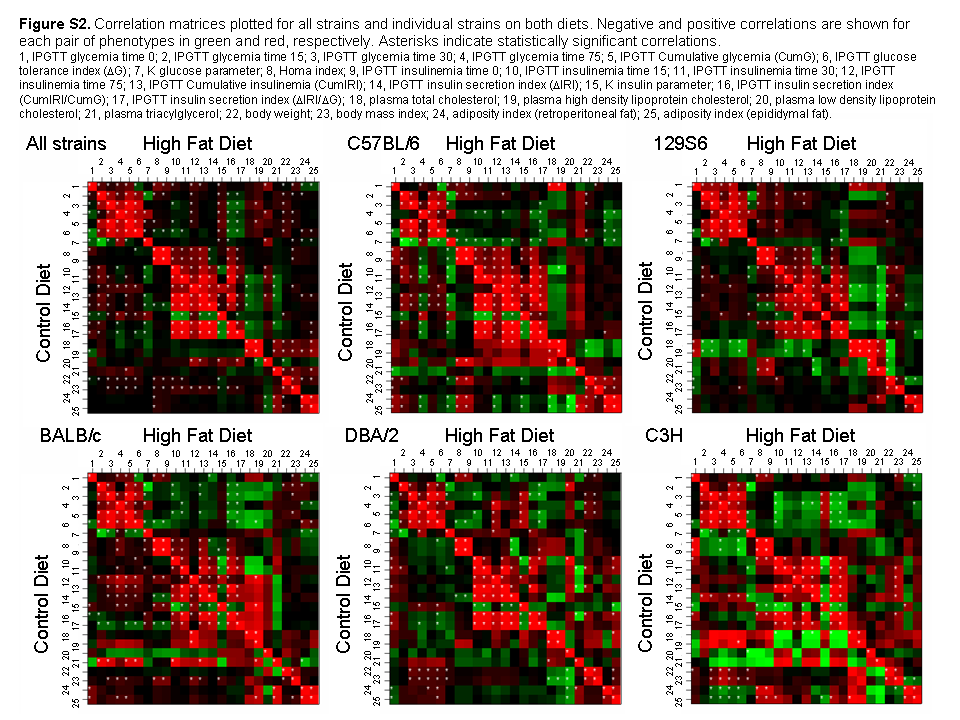

Supplement: Figure S2 — (0.62 MB TIF) [file pone.0001668.s002.tif]

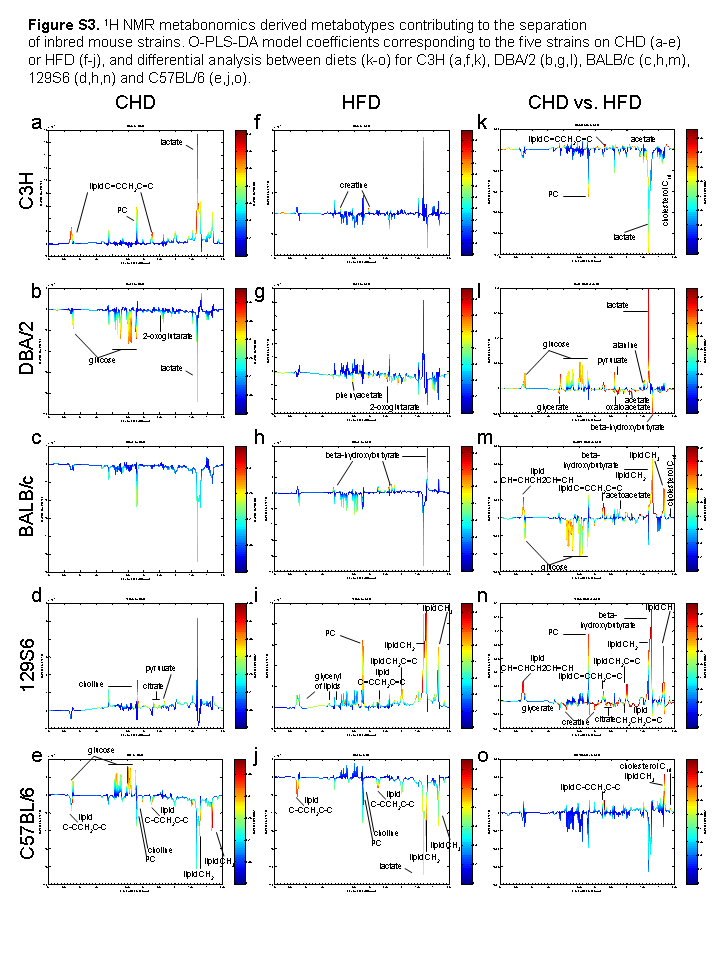

Supplement: Figure S3 — (0.14 MB TIF) [file pone.0001668.s003.tif]
